# 810. Severe Burn Disrupts Tissue-specific Clock Gene Expressions

**DOI:** 10.1093/jbcr/irag033.188

**Published:** 2026-04-06

**Authors:** Julia Kleinhapl, Rito Valdez, Steven E Wolf, Juquan Song

**Affiliations:** Division of Burn and Trauma Surgery, Department of Surgery, University of Texas Medical Branch, Galveston, Galveston, TX; University of Texas Medical Branch, Galveston, TX; Division of Burn and Trauma Surgery, Department of Surgery, University of Texas Medical Branch, Galveston, Galveston, TX; University of Texas Medical Branch, Galveston, TX

## Abstract

**Introduction:**

Severe burns trigger systemic inflammation and a hypermetabolic response. The impact on circadian biorhythms has not been determined. Clock gene-regulated circadian rhythms maintain body homeostasis under physiologic conditions and are affected in disease. We posit that severe burn disrupts tissue-specific clock gene expression. Our aim was to examine clock gene expression in blood and muscle in the acute phase after severe injury.

**Methods:**

Twenty-four adult male rats were randomized to 30% total body surface area (TBSA) scald burn or sham. Procedures were performed at 09:00, and animals were housed under regular light cycle (lights on 06:00-18:00). On the next day, animals were sequentially euthanized at 12:00 (+27 h after burn), 18:00 (+33 h), 00:00 (+39 h), and 06:00 (+45 h). EDTA blood was collected for isolation of peripheral blood mononuclear cells (PBMCs). Gastrocnemius muscle tissue was harvested, and total RNA was extracted from both tissues. Expression of PER1, PER2, CRY1, CRY2, Clock, and ARNTL was measured by TaqMan qPCR and expressed as fold change. The housekeeping gene was 18 s rRNA. Two-way ANOVA was performed in SigmaPlot 15.

**Results:**

Clock gene expressions showed the expected circadian rhythm in PBMCs from sham rats, with increased expression of Clock, ARNTL, CRY1, CRY2, PER1 and PER2 by over 1.5-2-fold during the daylight period, peaking at 18:00 (+33 h after sham) (*p*<.05). Expression decreased at night.

In PBMCs, burn caused overall suppression of Clock, CRY1, CRY2, and PER1 expression (main effect *p*<.05), and a marked late drop in PER2 at 39-45 h after burn (*p*<.05) compared with sham rats (Fig. 1). ARNTL and PER2 gene expression remained elevated at 18:00 (+33 h after burn) compared with the other time points following burn (*p*<.05).

In muscle tissue, burn significantly suppressed PER2, CRY1 and CRY2 (*p*<.05) compared with shams. In sham rats, peak PER1 expression was detected at 18:00 (+33 h) (5.586 ± 0.932 vs. 0.867 ± 0.932 at 12:00 (+27 h)), *p*<.05) and peak PER2 at 06:00 (+45 h) (26.301 ± 3.617 vs. earlier times, *p*<.05). Conversely, in burned rats, PER1 and PER2 peaked at 12:00 (+27 h), at 2.803 ± 0.932 and 2.388 ± 3.627, respectively. Clock and ARNTL were not detected consistently in muscle tissue.

**Conclusions:**

Our results indicate that tissue specific clock gene expression is inhibited after severe burn, while clock gene expression in sham groups showed expected circadian course.

**Applicability of Research to Practice:**

The circadian clock plays a key role in modulating muscle growth and maintenance of physiologic function. Understanding the correlation between post-burn circadian regulatory mechanisms may support the development of novel therapeutic strategies.

**Funding for the study:**

Remembering the 15.

